# The activation of GABAergic neurons in the hypothalamic tuberomammillary nucleus attenuates sevoflurane and propofol-induced anesthesia in mice

**DOI:** 10.3389/fphar.2023.1153735

**Published:** 2023-06-22

**Authors:** Jing Liu, Xiao Liu, Wen-Yu Zhou, Jun Gan, Jie Wang, Qi Zhang, Jun-Liang Li, Zhong-Shan Shen, Yue-Ying Zhang, Qiong-Yao Tang, Zhe Zhang

**Affiliations:** ^1^ Jiangsu Province Key Laboratory of Anesthesiology, Xuzhou Medical University, Xuzhou, Jiangsu, China; ^2^ Jiangsu Province Key Laboratory of Anesthesia and Analgesia Application Technology, Xuzhou Medical University, Xuzhou, Jiangsu, China; ^3^ NMPA Key Laboratory for Research and Evaluation of Narcotic and Psychotropic Drugs, Xuzhou Medical University, Xuzhou, Jiangsu, China; ^4^ Department of Cell Biology and Neurobiology, Life Sciences College, Xuzhou Medical University, Xuzhou, Jiangsu, China; ^5^ Department of Anesthesia, The Affiliated Hospital of Xuzhou Medical University, Xuzhou, Jiangsu, China

**Keywords:** sevoflurane, propofol, hypothalamic tuberomammillary nucleus, gabaergic neurons, anesthesia

## Abstract

**Background:** The histaminergic neurons in the hypothalamic tuberomammillary nucleus (TMN) have been suggested to play a vital role in maintaining a rising state. But the neuronal types of the TMN are in debate and the role of GABAergic neurons remains unclear.

**Methods:** In the present study, we examined the role of TMN GABAergic neurons in general anesthesia using chemogenetics and optogenetics strategies to regulate the activity of TMN GABAergic neurons.

**Results:** The results indicated that either chemogenetic or optogenetic activation of TMN GABAergic neurons in mice decreased the effect of sevoflurane and propofol anesthesia. In contrast, inhibition of the TMN GABAergic neurons facilitates the sevoflurane anesthesia effect.

**Conclusion:** Our results suggest that the activity of TMN GABAergic neurons produces an anti-anesthesia effect in loss of consciousness and analgesia.

## Introduction

General anesthesia causes reversible loss of consciousness through diverse neural circuits (Franks, 2008). Unconsciousness rather than unresponsiveness and amnesia requires the inactivation of specific brain regions, which results in a loss of functional integration in particular areas (Alkire et al., 2008). Among the brain regions, the hypothalamic tuberomammillary nucleus (TMN) has been suggested to play a vital role in the sedative response to GABAergic anesthetics (Nelson et al., 2002). However, a subsequent study indicates the histaminergic neurons lesion or block H1 receptor facilitates isoflurane-induced anesthesia but not propofol, pentobarbital, and ketamine-induced anesthesia (Luo and Leung, 2011). Also, another study stated that the histaminergic TMN could not play a central role in anesthesia because genetically removing ionotropic GABAa receptors in histaminergic neurons does not affect the emergence time of propofol-induced loss of righting reflex (Zecharia et al., 2012). Furthermore, several studies using chemogenetic and optogenetic approaches also give controversial results for the role of TMN histaminergic neurons in arousal state maintenance. Some studies report the chemogenetic inhibition of histaminergic neurons by hM4Di decreases wakefulness and increases NREM sleep, while acute optogenetic silencing of histamine neurons using Arch3.0 promotes NREM sleep but not REM sleep (Fujita et al., 2017; Yu et al., 2019). However, another group reported inhibiting histaminergic neurons with ArchT during wakefulness does not impact NREM sleep (Venner et al., 2019).

Although many studies focus on the role of histaminergic neurons in the TMN in arousal and anesthesia, the role of GABAergic neurons in TMN was largely ignored. Furthermore, an earlier study showed that ablation of the Vgat gene in histaminergic neurons in the TMN produced hyperactive mice, implicated in the co-transmission of GABA and histamine to control the awake state (Yu et al., 2015). But this result was challenged by another report that genetic deletion of the GABA synthesis enzyme in TMN does not alter sleep-wake quantities, which also showed that VGAT and histidine decarboxylase (HDC) were co-expressed only in a small fraction of TMN HDC neurons (Venner et al., 2019). Thus, the impact of TMN GABAergic neurons in general anesthesia needs further investigation. Early studies have demonstrated TMN neurons are heterogeneous, including adenosine deaminase (ADA), HDC, and glutamate decarboxylase (GAD) neurons (Staines et al., 1986). In TMN GAD-positive neurons, the expression of GAD67 mRNA was robust, but no labeling for GAD65 mRNA was evident (Esclapez et al., 1993). A recent study found the GABAergic neurons from TMN may project to lateral habenula in zebrafish (Ramaswamy et al., 2020). But in general, the projection and function of the TMN GABAergic neurons in mice remain unclear.

Furthermore, some studies found ascending GABAergic neurons may promote cortical activation and maintain an arousal state (Brown and McKenna, 2015). But whether GABAergic neurons in TMN play a similar role needs further investigation. To address the role of TMN GABAergic neurons in loss of consciousness in anesthesia, we injected the AAV-hsyn-DIO-chR2-mcherry or AAV-hsyn-DIO-hM3q/hM4i virus in VGAT-Cre transgenic mice to manipulate the activity of GABAergic neurons in TMN by light or by CNO to examine its role in maintaining the arousal state under propofol and sevoflurane anesthesia. Our results suggest that activation of TMN GABAergic neurons produces an anti-anesthesia effect in loss of consciousness and analgesia.

## Materials and methods

### Animals

This study followed the guidelines described in the Guide for the Care and Use of Laboratory Animals in China (No. 14924, 2001) and was approved by the Animal Care and Use Committees of Xuzhou Medical University. Adult male VGAT-Cre mice were obtained from the Jackson Laboratory. Mice were housed in standard chambers within an SPF laboratory animal room (12/12-h light/dark cycle, lights on between 07:00 and 19:00; 23°C ± 2°C; relative humidity: 55% ± 2%) with free access to food and water.

### Chemicals

Sevoflurane was purchased from Heng Rui Pharmaceutical Co. Ltd. (China). Continuous administration of 2% sevoflurane, carried by oxygen with a flow rate of 1 L/min, was used for behavioral tests and EEG recording. Propofol was obtained from Life Technologies Corporation (United States). 1% propofol was diluted to 0.1% or 0.2% with 5% glucose for use.

### Surgery and stereotaxic injections

Mice were anesthetized with sodium pentobarbital (50 mg/kg, i.p.) and secured into a stereotaxic frame. Burr holes were drilled immediately above the TMN. The virus was injected into the TMN (anteroposterior (AP) = −2.4 mm; lateral (ML) = ± 0.75 mm; dorsoventral (DV) = −5.4 mm) through a glass micropipette with a tapered tip (10–20 μm), which was connected to the air compression system. Then, the injections were implemented using an electronic air compression system. After injection, the pipette was withdrawn after 5 min of waiting (Liu et al., 2017). AAV vectors (AAV-hSyn-DIO-hM3Dq-mCherry, AAV-hSyn-DIO-hM4Di-mCherry, AAV-hSyn-DIO-hChR2-mCherry, AAV-hSyn-DIO-mCherry-WPRE-pA) were purchased from Taitool company (Shanghai, CHINA). The adult male mice were divided into hM3Dq, hM4Di, ChR2, and mCherry control groups based on the different viruses injected. Each AAV vector was bilaterally microinjected into the TMN region at a volume of 80 nL, respectively (Virus titer was 1.32E ×10^13^ V.G/mL).

### EEG and optical fiber implants

Two weeks after the virus injection, the mice were implanted with electrodes for EEG recordings. Four skull nails anchored on the skull surface were used to fix the accessory for EEG recording to transmit the electrical signals of the cerebral cortex to the preamplifier. For optimal EEG alignment, the front edge of the implant should be placed 3.0–3.5 mm anterior of the bregma. In this configuration, all four screws will rest in the cerebral cortex region of a fully-grown mouse. The left electrode near the bregma is the reference electrode. For the optogenetic stimulation of the TMN, the optical fibers (diameter: 200 μm, length: 5.5 mm, numerical aperture: 0.37; Hangzhou Yingke Biotechnology Co., Ltd., China.) were implanted over the TMN (AP: −2.4 mm, ML: ± 0.75 mm, DV: −5.2 mm). Each optical fiber was guided into position using a stereotaxic atlas and glued into place using a mixture of dental cement and cyanoacrylate glue before the accessory was assembled into the layer of dental cement.

### EEG recording

The Electroencephalogram (EEG) was used to monitor the anesthesia depth in all groups (Purdon et al., 2015). After 2 weeks following the AAV injection, mice were implanted with the accessory for EEG recordings. The multichannel signal acquisition system (Pinnacle Technologies, United States) was used to acquire EEG signals. The EEG signals were collected at 400 Hz and bandpass filtered at 0.5–30 Hz. The EEG showed that the period of anesthesia maintenance during CSSGA (continuous, steady-state general anesthesia) was selected for analysis. Relative powers in the different frequency bands were computed by averaging the signal power across the frequency range of each band (δ: 1–4 Hz, θ: 4–8 Hz, α: 8–12 Hz, β: 12–25 Hz). EEGs were analyzed using the Chronux toolbox in MATLAB 2016a (MathWorks, Cambridge, United States) to estimate the depth of general anesthesia (Liu et al., 2021).

### Behavioral tests

The mice’s behavior responses to general anesthetics were examined by examining the latency of LORR, LOTW, and RORR. LORR (loss of right reflex) latency time was determined by the interval between the onset of the administration of general anesthetic and when the mice manifested LORR for more than 5 s. LOTW (loss of tail withdrawal upon pinch) latency time was determined by the interval between the onset of the administration of the general anesthetics and the time at which the mice manifested LOTW. The RORR (recovery of righting reflex) time was determined as interval time began from sevoflurane off to when the mice regained a prone position.

To examine the LORR induced by sevoflurane, we placed the mouse in a small rectangle induction box with the tail left outside of the box through a tiny hole on the sidewall of the induction box. After administering sevoflurane, LORR was examined by turning box 90° every 15 s to obtain the latency time. LOTW was examined every 30 s by pinching the tails of mice with hemostatic forceps after the LORR appearance until the mice lost the withdrawal reflex upon pinching.

Immediately after the cessation of sevoflurane, the mice were gently taken out from the induction box, placed supine on a soft pad, and exposed to room air for recovery. The duration between the cessation of sevoflurane and when the mice regain the normal prone position was defined as the RORR time.

The mouse was placed in the induction box immediately after the intravenous propofol injections (i.v) for anesthesia. RORR was defined as the interval from when the mice manifested LORR to the time of regaining a prone position.

The dose-response curves of sevoflurane or propofol were measured as follows: each mouse in different groups was given a fixed concentration of sevoflurane or propofol to observe the numbers of mice that resulted in LORR or LOTW. The mice given sevoflurane were observed 6 min after a fixed dose inhalation, while the mice with a dose administration of propofol were observed after an i.v injection. Forty-8 h later, a higher sevoflurane concentration was given to watch and count the mice resulting in LORR and LOTW. The process was repeated until the dose reached a concentration that resulted in all mice in LORR or LOTW. The sevoflurane concentrations used were (in atm%) 1, 1.25, 1.35, 1.45, 1.55, 1.65, 1.75, 1.85, and 2, respectively. The gradient propofol doses used were (in mg/kg) 5, 6, 7, 8, 9, 10, 11, 12,13, 14, 15, and 16, respectively. When the dose is below 12 mg/kg, the propofol was diluted to 1% (m/V). When the dose is 12 mg/kg or above, the propofol was diluted to 2% (m/V). The dose-response equation fitted the dose-effect curve using GraphPad Prism 8.0.

A double-blind observation strategy was used to observe the anesthesia behavior of virus-injected mice. The researcher who injected the virus provided mice to the researcher who observed the behavior. The researcher who observed the mice’s anesthesia behavior did not know the mice he/she used were injected with the effective virus or control virus.

### Chemogenetic activation/inhibition

The adult male mice were divided into hM3Dq, hM4Di, and mCherry control group based on the different viruses injected. Clozapine N-oxide (Saint Louis, MO, United States) (1 mg/kg, i.p) was injected for 1 hour before behavior testing and EEG recording (Liu et al., 2017). Gradient concentrations of sevoflurane were used to detect each mouse’s dose results in LORR and LOTW. Meanwhile, the latency of LORR and the RORR time during inhalation of 2% sevoflurane were observed. EEG recording was performed during CSSGA (continuous, steady-state general anesthesia) with 2% sevoflurane. Gradient propofol concentrations were used to detect each mouse’s dose that caused LORR. The RORR time and EEG were observed under 20 mg/kg of intravenous propofol injections. These indicators are related to the sensitivity of mice to inhaled and intravenous anesthetics. Each animal was tested multiple times with at least a 5-day washout between experiments to reduce the number of mice used.

### Optogenetic activation

The adult male VGAT-Cre mice were divided into the ChR2, and the mCherry control virus groups based on the different viruses (AAV-hSyn-DIO-hChR2-mCherry, AAV-hSyn-DIO-mCherry) injected. A 473 nm laser performed the optical activation at 20 Hz for 10 ms. Each stimulation cycle was 10 s on and 20 s off. In observing the anesthesia behavior under gradient doses of propofol or sevoflurane**,** the average concentration results in LORR and LOTW, the latencies induced to LORR, and RORR time were measured. The EEGs were recorded during a continuous optogenetic stimulation process.

### Perfusion and immunohistochemistry

After the behavioral testing and EEG recording, the brains were removed, post-fixed in 4% PFA overnight, and incubated in 30% sucrose in PBS at 4°C until they sank. Coronally sectioned slices (30 µm) were cut on a cryostat (Leica CM 1950). Sections were incubated with rabbit anti-GABA (Invitrogen, United States) primary antibodies overnight at 4°C. Slides were rinsed with distilled water and labeled with Alexa Fluor 594 goat IgG (Invitrogen, United States) for 2 h. Images were captured using a BX51WI microscope (Olympus, Japan).

## *In vitro* electrophysiological experiments

### Verification of the effect of optogenetic stimulation

Acute, coronal brain slices (300 μm) containing the TMN were collected from AAV-DIO-ChR2 injected VGAT-Cre mice 4 weeks after injection for *ex vivo* electrophysiological recordings. Briefly, mice were decapitated following isoflurane anesthesia, and the brains were removed and immersed in ice-cold modified artificial cerebrospinal fluid (ACSF) saturated with 95% O_2_ and 5% CO_2,_ and that contained the following (in mM): 130 NaCl, 26 NaHCO_3_, 1 CaCl_2_, 0.5 MgCl_2_, 3.5 KCl, 10 glucose, 1.25 NaH_2_PO_4_ (Wang et al., 2018; Li et al., 2020). The tissue block containing the TMN was then mounted in a vibrating microtome (Leica VTS-1000) and coronal slices were prepared. Slices were transferred to a holding chamber containing ACSF, incubated for 30 min at 33°C, and subsequently maintained at RT for 30 min before recordings. Slices were placed in a Warner Series 20 recording chamber (Warner Instruments) mounted on the fixed stage of an Olympus DX51 microscope. Slices were fully submerged and continuously perfused at a rate of 1–2 mL/min oxygenated ACSF. Before patch clamping, a 200 μm fiber-optic cable connected to a 473 nm laser (Shanghai Lasers) was placed at the external capsule aimed at the TMN. 473 nm light was delivered at 20 Hz. Whole-cell current-clamp techniques were used to measure action potential firing with the internal solution containing (in mM) 20 KCl, 100 K-gluconate, 10 HEPES, 4 ATP, 0.5 GTP, and 10 phosphocreatine (Liu et al., 2017; Wang et al., 2018). Data were acquired with an EPC10 amplifier and analyzed by pulse fit software.

### Verification of chemogenetic activation

Brain slices containing TMN neurons were used as described above without light, but with CNO (5 μM bath applied). The slices were patched in the current-clamp configuration.

### Statistical analysis

The data are presented as mean ± SEM. Statistical analysis was performed with GraphPad Prism, version 8.0. Statistical significance was assessed using Student’s t-test to compare two groups. One-way analysis of variance (ANOVA) for three groups. The EEG signal was analyzed with MATLAB. In all cases, *p* < 0.05 was considered significant. The dose-response curves of sevoflurane and propofol were fitted by the dose-response equation. The equation is described as $y=A1+\frac{A2-A1}{1+10^{\left(LOGx0-x\right)p}}$ (1).

A1 is the percent of mice in the anesthesia state with a minimal concentration of general anesthetic, while A2 defines the percent of mice in the anesthesia state with the maximal concentration of general anesthetic. LOGx0 is the anesthetic concentration that caused 50% of mice in anesthesia (EC50). P defines the slope of the curve. GraphPad Prism8.0 made the comparison of the dose-response curve. Statistical analysis of the slope curve used Extra Sum-of-squares F-test.

## Results

### Chemogenetic activation of TMN GABAergic neurons attenuates sevoflurane-induced anesthesia

**FIGURE 1:**
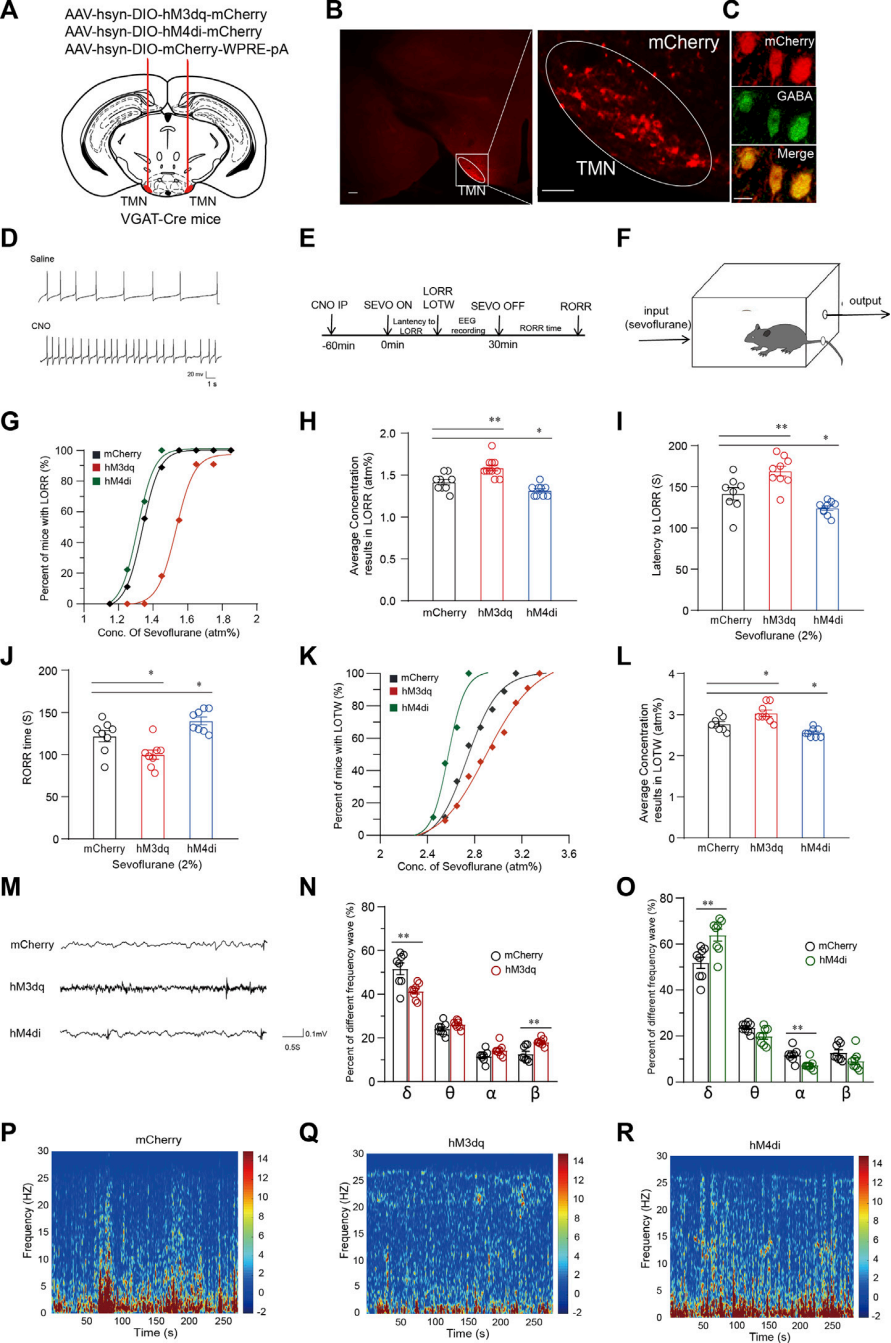
Chemogenetic manipulation of TMN GABAergic neurons alters induction, maintenance and emergence from sevoflurane anesthesia. **(A)** Schematic representation showing the location of virus injection tuberomammillary nucleus (TMN) in a coronal brain section of VGAT-Cre mice. **(B)** The representative fused mcherry fluorescence showed effective receptor expression in TMN (scale bar, 100 μm). The co-localization of mCherry and GABA is shown in **(C)**. **(D)** Compared with saline, bath application of CNO increased firings in an hM3Dq-mCherry neuron. **(E)** The timeline of experimental manipulation covers the process of sevoflurane anesthesia induction time (LORR) and emergence time (RORR), and EEG recording. **(F)** Schematic representation of induction box in which the sevoflurane concentration was maintained to the indicated level for mice anesthesia. **(G)** The sevoflurane dose-response curve of LORR fitted by the dose-response formula in hM3Dq, hM4Di and control group, respectively. **(H)** The averaged concentration of sevoflurane results in LORR in the hM3Dq, hM4Di, and control groups. **(I)** The latency to LORR of hM3Dq, hM4Di, and control groups, respectively, under 2% sevoflurane induction. **(J)** The RORR time of hM3Dq, hM4Di and control groups, respectively, under 2% sevoflurane induction. **(K)** The sevoflurane dose-response curve of LOTW fitted by the dose-response function in hM3Dq, hM4Di and control group, respectively. **(L)** The averaged concentration of sevoflurane results in LOTW of half mice in hM3Dq, hM4Di, and control groups. **(M)** Representative 5 s EEG traces of hM3Dq, hM4Di, and mCherry mice, respectively, during general anesthesia under 2% sevoflurane. **(N)** The comparison of waveforms proportion in the EEG of hM3Dq group mice with the wave proportion of EEG obtained in the mCherry group during continuous inhalation of 2% sevoflurane for 5 min. **(O)** The comparison wave components proportion in EEG of hM4Di group mice with the proportion in EEG of control group mice. **(P-R)** The 5-min EEG power density spectra color-coded on a logarithmic scale [dB] of the mCherry control mice group **(P)**, hM3Dq mice group **(Q)**, and hM4Di mice group **(R)**. Mean ± SEM. * p < 0.05, ** p < 0.01, *** p < 0.001, **** p < 0.0001.

To explore whether manipulating the activity of TMN GABAergic neurons affects loss of consciousness induced by general anesthetic, we first utilized the DREADD (Designer-receptors exclusively activated by designer drugs) approach to express the hM3Dq receptor or hM4Di receptor into the TMN GABAergic neurons by bilateral injection of virus (AAV-hSyn-DIO-hM3Dq-mCherry or AAV-hSyn-DIO-hM4Di-mCherry) into the TMN of VGAT-Cre mice. The control group of mice was injected with the AAV-hSyn-DIO-mCherry-3Flag-WPRE-SV40pA virus (Figure 1A). The red fluorescence signal of the fused cherry protein was detected by a fluorescent microscope validated the receptors’ expression in TMN (Figure 1B). Double immunofluorescence staining revealed that mCherry systematically co-localized with GABA in the TMN (Figure 1C). *In vitro* brain slice recording verified that hM3Dq expressed neurons fired more frequently than the neurons without hM3Dq expression after CNO application (Figure 1D). Then, the loss of righting reflex (LORR), loss of tail-withdraw reflex (LOTW), and the recovery of the righting reflex (RORR) induced by sevoflurane were detected in the groups of mice after chemogenetic activation or inhibition of TMN GABAergic neurons. In the meantime, the mice’s anesthesia depth was estimated by EEG recording (Figures 1E,F).

The sevoflurane dose-response curves were generated using the percentage of mice that manifested LORR at the measured concentrations in their respective groups. Results showed the dose-response curve for the LORR effects of sevoflurane in the hM3Dq group was rightward-shifted (n = 11, *p* < 0.0001) compared with the curve in the mCherry group (n = 9), whereas the significant leftward-shifted of the curve was observed in the hM4Di group (n = 9, *p* = 0.007) (Figure 1G). Consistently, the average concentration of sevoflurane that caused LORR in the CNO-activated hM3Dq group (1.59 ± 0.03 atm%) (atmosphere pressure) was significantly higher than the concentration in the control virus injected group (1.42 ± 0.03 atm%, *p* = 0.0013). In contrast, the average sevoflurane concentration that resulted in LORR of mice in the hM4Di group (n = 9, 1.32 ± 0.02 atm%) was significantly lower than the concentration in the control group (*p* = 0.038) (Figure 1H). Furthermore, upon 2% sevoflurane inhalation, the average LORR latency in the hM3Dq group was 27.6 s longer than the control group’s (141.5 ± 7.07 s vs. 169.11 ± 5.68 s; *p* = 0.005). In contrast, the latency to LORR in the hM4Di group was significantly decreased by approximately 12% (141.5 ± 7.07 s vs. 124.1 ± 2.66 s; *p* = 0.04) (Figure 1I). Similarly, after turning off the sevoflurane pump, the average RORR time in the hM3Dq group was significantly shorter than in the mCherry group (n = 8, 121.88 ± 6 s vs. 99.88 ± 5 s, *p* = 0.02). In contrast, the RORR time in the hM4Di group was considerably longer than the RORR time of the control group (n = 8, 121.88 ± 6 s vs. 140 ± 4 s, *p* = 0.04) (Figure 1J). Subsequently, the analgesic effect of sevoflurane was also examined by tail clipping experiment. We observed a rightward shift of the LOTW (Loss of tail withdrawal) sevoflurane dose-response curve in the hM3Dq group. In the meantime, the average concentration that resulted in LOTW in the hM3Dq group was statistically higher than the concentration in the control mice group, indicating that chemogenetic activation of the TMN GABAergic neurons significantly reduced the analgesic effect of sevoflurane (2.78 ± 0.06 atm% vs. 2.83 ± 0.07 atm%; *p* = 0.012) (Figures 1K–L). In contrast, inhibition of the TMN GABAergic neurons (hM4Di group) (n = 8) significantly leftward shifted the LOTW sevoflurane dose-response curve of the mice and significantly decreased the average sevoflurane concentration of causing LOTW (2.78 ± 0.06 atm% vs. 2.56 ± 0.04 atm%; *p* = 0.043) (Figures 1K–L). Thus, the chemogenetic activation of TMN GABAergic neurons attenuated the loss-of-conscious and the analgesic effect of sevoflurane, whereas inhibiting the activation of TMN GABAergic neurons facilitated sevoflurane’s analgesic and the loss-of-conscious induction effect.

To further examine how the activity of TMN GABAergic neurons alters the anesthesia state induced by sevoflurane, we performed EEG recording in the cortex of the mice during CSSGA (continuous, steady-state general anesthesia) under 2% sevoflurane. The results showed activation or inhibition of TMN GABAergic neurons also exerted a significant change in the power spectrum during CSSGA with 2% sevoflurane (Figure 1M). While activating TMN GABAergic neurons caused the decreased energy of the δ wave (n = 8, 52% ± 2.3% vs. 41% ± 1.2%; *p* < 0.01) and an increase in the energy of the β wave (n = 8, 12% ± 1.2% vs. 18% ± 0.5%; *p* < 0.01) (Figure 1N), indicating a reduced depth of anesthesia (Figures 1P–R). Inhibition of TMN GABAergic neurons caused the increased energy of the δ wave (n = 8, 52% ± 2.3% vs. 64% ± 2.5%; *p* < 0.01) and decreased energy of the α wave (n = 8, 14% ± 1% vs. 7% ± 1%; *p* < 0.01) (Figure 1O), indicating the cortex entered a deeper anesthesia state (Figures 1P–R) (Luo et al., 2018). Thus, the EEG data proved that chemogenetic activation of TMN GABAergic neurons drove cortical arousal during general anesthesia with sevoflurane.

### Chemogenetic activation of TMN GABAergic neurons attenuates the efficacy of propofol-induced general anesthesia

**FIGURE 2:**
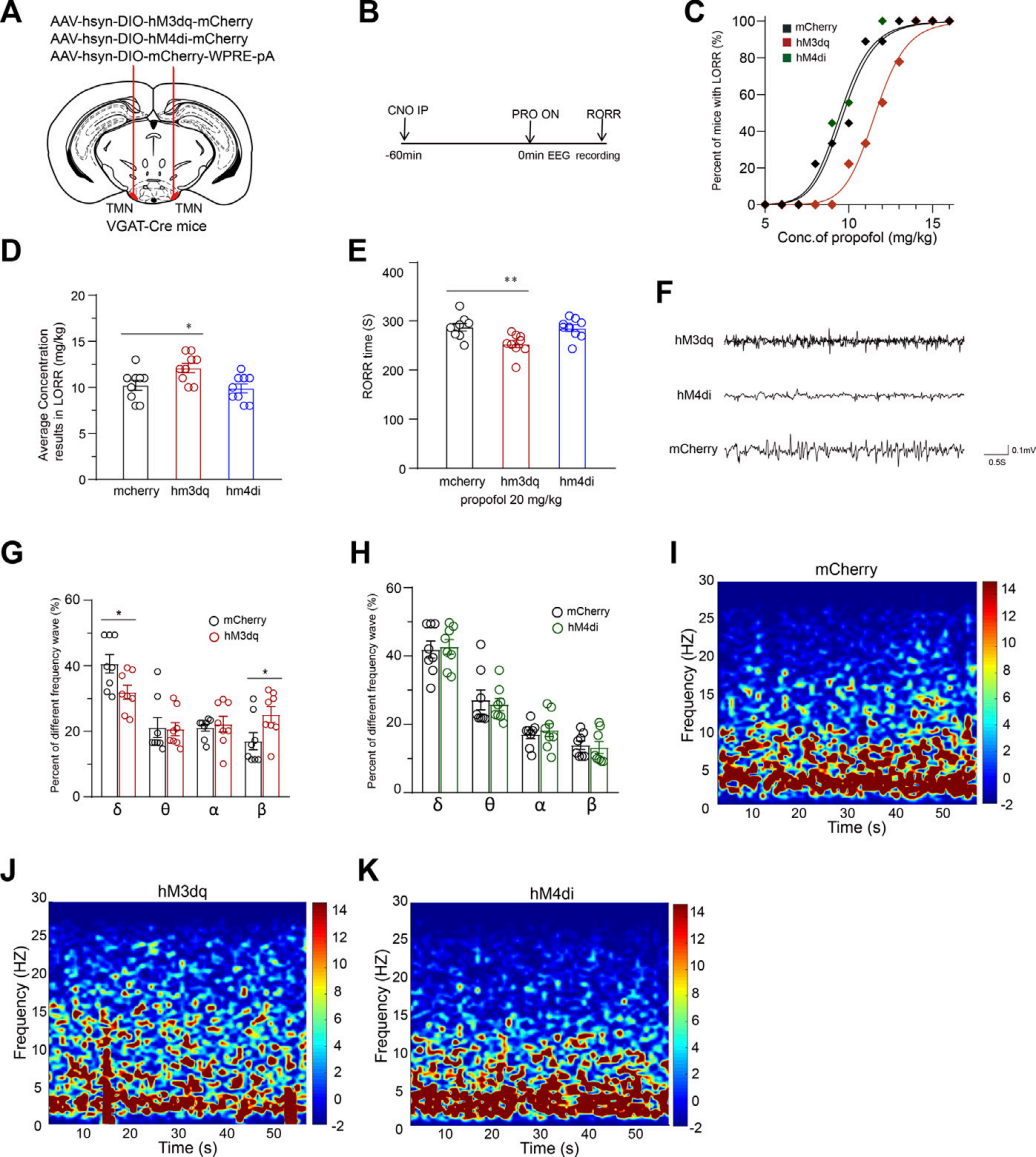
Chemogenetic manipulation of TMN GABAergic neurons promotes emergence from propofol anesthesia. **(A)** Schematic representation for chemogenetic stimulation of TMN GABAergic neurons. **(B)** The sequential experimental timeline covers the chemogenetic activation of TMN GABAergic neurons, the beginning of propofol application to the end of emergence time (RORR), and EEG recording. **(C)** The LORR propofol dose-response curves of hM3Dq, hM4Di, and mcherry control groups. **(D)** The averaged concentration of propofol that caused LORR of mice in the hM3Dq group, hM4Di group, and control group. **(E)** The RORR times of mice induced by propofol in the hM3Dq group, hM4Di group, and mcherry control group were induced by intravenous injection of 20 mg/kg of propofol after CNO activation. **(F)** Representative traces of EEG during anesthesia were recorded from hM3Dq mice, hM4Di mice, and mCherry mice, respectively. **(G–H)** Comparison of frequential waveform components in 60-s EEG during anesthesia induced by propofol recording from mice in the control group with the components of EEG of mice in the hM3Dq and hM4Di groups, respectively. **(I–K)** Averaged 60-s mice EEG power density spectra of the mCherry control group **(I)**, hM3Dq group **(J)**, and hM4Di group **(K)**. Mean ± SEM, * p < 0.05, ** p< 0.01.

To further test how the activity of TMN GABAergic neurons influences propofol (Intravenous Injections, I.V, 20 mg/kg) anesthesia, we continuously used DREADD technology to modulate the activity of TMN GABAergic neurons (Figures 2A,B). The chemogenetic activation of TMN GABAergic neurons (n = 9) significantly rightward shifts the propofol-induced LORR dose-response curve compared to the curve in the mCherry control group (n = 9, *p* < 0.001). However, the chemogenetic inhibition of TMN GABAergic neurons (hM4Di group) (n = 9) does not induce significant LORR dose-response curve shift compared to the mcherry group (Figure 2C). Consistently, a significant difference in the dose of propofol resulted in LORR in 50 percent of mice between the hM3Dq group and the control group was observed (10.22 ± 0.52 mg/kg vs. 12.11 ± 0.48 mg/kg; *p* = 0.02), whereas no significant difference between the hM4Di group and the mcherry group (Figure 2D). Meanwhile, the RORR time in the hM3Dq group was significantly reduced by approximately 35 s compared with the time of the mCherry group (287 ± 7 s vs. 252 ± 6.8 s; *p* = 0.005), whereas the RORR time in the hM4Di group was not significantly altered during the anesthesia of 20 mg/kg propofol (Figure 2E). Thus, the chemogenetic activation of TMN GABAergic neurons reduced the effect of propofol for inducing unconsciousness, but chemogenetic inhibition of TMN GABAergic neurons did not alter the anesthesia effect of propofol.

We recorded EEG over the corresponding cortical area during CSSGA with 20 mg/kg propofol intravenous injection. The analyzed results showed the activation of GABAergic neurons in TMN caused a decreased ratio of δ waves (n = 8, 41% ± 2.6% vs. 32% ± 2%; *p* = 0.03), whereas a ratio increase of the β waves (n = 8, 17% ± 2% vs. 25% ± 2%; *p* = 0.04) (Figures 2F–K). However, inhibition of GABAergic neurons in TMN did not cause a significant difference in the wave patterns in the EEG and power spectrum from those values in the mCherry group (Figures 2F–K). The EEG data suggest that the activation but not inhibition of TMN GABAergic neurons drives cortical arousal state alteration during CSSGA with propofol.

### Optogenetic activation of TMN GABAergic neurons attenuates sevoflurane-induced anesthesia and analgesic effect

**FIGURE 3:**
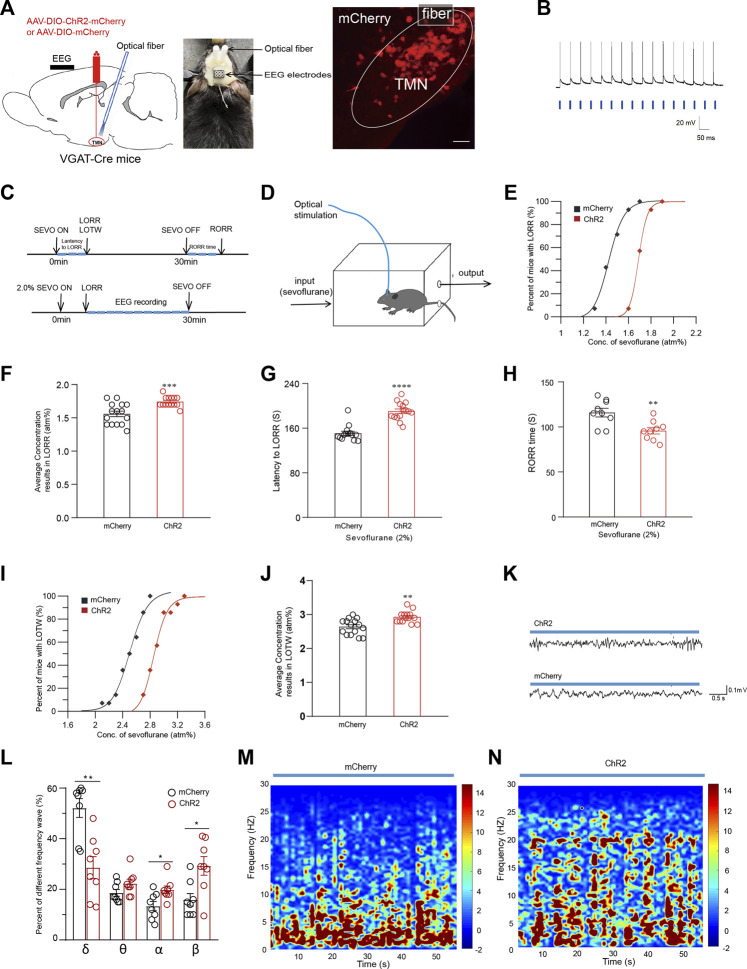
Optical activation of TMN GABAergic neurons alters the general anesthesia state of sevoflurane anesthesia. **(A)** Left: Diagram of sagittal brain section showing the location of virus injection and fiber embedding; Right: The fluorescence of mcherry indicates the TMN GABAergic neurons were infected by the ChR2-mCherry virus (scale bar, 50 μm). **(B)** Blue light-evoked action potentials were recorded from a mCherry positive neuron in the TMN in response to photostimulation (20 Hz, denoted by blue bars). **(C)** Protocol for optogenetic activation during sevoflurane anesthesia. **(D)** Schematic induction box in which the mice were under the given sevoflurane concentration levels. The mice were constantly optically stimulated (activation, 473 nm, 20 Hz, and 10 ms duration) every 10 s with 20-s intervals from the beginning to the end of sevoflurane inhalation. **(E)** The dose-response curves plotted from the proportion of LORR mice under graded sevoflurane in ChR2 and mCherry control group. **(F)** The average sevoflurane concentration caused mice’s LORR in ChR2 and mcherry control groups. **(G–H)** The average latency to LORR **(G)** and RORR time **(H)** of the mice exposed to 2% sevoflurane in the ChR2 and mcherry control group, respectively. **(I)** The sevoflurane dose-response curves caused LOTW in mice in ChR2 and mcherry control groups. **(J)** The average sevoflurane concentration caused LOTW of mice in the optical stimulation of ChR2 and mcherry control group. **(K)** Representative traces of the EEG of mice in optical stimulation of ChR2 and mcherry control group, respectively. **(L)** Comparison of the frequency distribution of waveform in the EEG of mice in optical stimulation of ChR2 and mcherry control group during 2% sevoflurane exposure. **(M-N)** The heatmap of the frequency distribution of waveform in 60-s EEG with optical stimulation of mcherry control **(M)** and ChR2 group **(N)**, respectively. Mean ± SEM.* p <0.05, ** p <0.01, *** p <0.001, **** p <0.0001.

To further estimate the role of TMN GABAergic neurons in the loss of consciousness induced by general anesthetic, we also utilized the optogenetic approach to activate TMN GABAergic neurons. The AAV-hsyn-DIO-hChR2(H134R)-mCherry virus (ChR2 group) or its control AAV-hsyn-DIO-mCherry (mCherry group) were injected into the TMN of VGAT-Cre mice. Meanwhile, an optical fiber was implanted 0.2 mm above the injection site to facilitate the light activation of TMN GABAergic neurons (Figure 3A). Three weeks later, patterned light reliably generated corresponding action potentials in the TMN neurons in the cultured brain slices (Figure 3B). Then a 473 nm blue light was given through the fiber to activate TMN GABAergic neurons. At the same time, the mice were placed into a closed rectangle induction box to observe LORR, LOTW, and RORR and monitor the anesthesia state by EEG (Figures 3C,D) under sevoflurane anesthesia.

The percentage of mice exhibiting LORR and LOTW versus the applied sevoflurane concentration was plotted as sevoflurane dose-response curves. The dose-response curve for the LORR effect of sevoflurane in the ChR2 group was rightward-shifted compared with the curve in the mCherry group (Figure 3E). Consistently, the average concentration of sevoflurane that causes 50% of mice exhibiting LORR in the ChR2 group was significantly increased compared with the mCherry group (n = 14, 1.59 ± 0.04 atm% vs. 1.75 ± 0.02 atm%, *p* < 0.001) (Figure 3F). Meanwhile, under 2% sevoflurane inhalation, the average latency to LORR in the ChR2 group was significantly longer than the latency in the mCherry group (n = 14, 150.9 ± 4 s vs. 190.5 ± 4 s, *p* < 0.0001) (Figure 3G). Consistently, the average RORR time in mice of the ChR2 group was significantly shorter than that of the mCherry group (n = 9, 116 ± 4.5 s vs. 96 ± 3.9 s, *p* = 0.0038) (Figure 3H). Subsequently, the analgesic effect of sevoflurane was also examined by the loss of tail withdrawal (LOTW) response to clipping. Results showed the sevoflurane dose-response curve for causing LOTW in the ChR2 group was also significantly rightward-shifted compared with the curve in the mCherry group (*p* < 0.001) (Figure 3I). The average sevoflurane concentration that causes LOTW in the ChR2 group was substantially higher than in the mCherry group (2.6 ± 0.06 atm% vs. 2.9 ± 0.04 atm%, *p* = 0.0019) (Figure 3J). These data indicate the optical activation of TMN neurons attenuates both the loss-of-conscious effect and the analgesic effect of sevoflurane.

During optical stimulation, there were also significant differences in the wave pattern and power spectrum in the EEG recording between the ChR2 group and the mCherry group during CSSGA with 2% sevoflurane. After 5 minutes of anesthetic administration, the EEG recordings during CSSGA with 2% sevoflurane were selected for the statistical analysis (Figure 3K). Compared with the mice in the mCherry group, the optogenetic activation of the TMN resulted in a decrease of δ-waves in EEG by approximately 22.54% (52% ± 3% vs. 29% ± 4%; *p* < 0.01) in the mice of the ChR2 group, whereas α and β waves in the EEG of mice in ChR2 group were marked increased (Figure 3L), indicating a reduced anesthesia depth (Figures 3M–N). Thus, the optogenetic activating of TMN GABAergic neurons attenuates both the loss of consciousness and the analgesic effect induced by sevoflurane.

### Optogenetic activation of TMN GABAergic neurons promotes arousal from propofol-induced anesthesia

**FIGURE 4:**
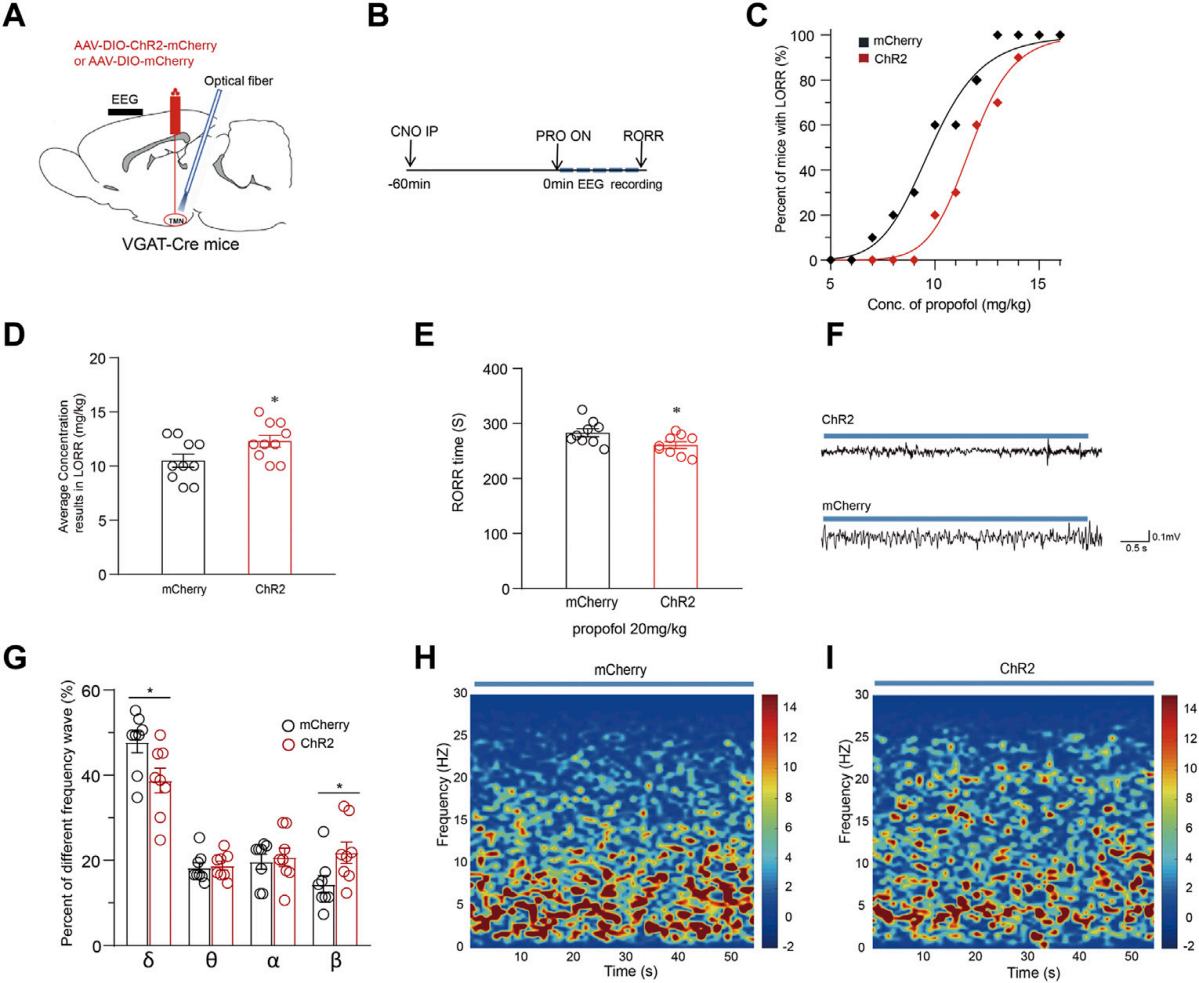
Optical activation of TMN GABAergic neurons alters the maintenance and emergence time from propofol anesthesia. **(A)** The diagram of the sagittal brain section shows virus injection and optical fiber embedding in the TMN of VGAT-Cre mice. **(B)** The sequential procedure of optogenetic activation and the propofol anesthesia behavior test process. **(C)** The propofol dose-response curve of LORR of mice with optical stimulation of VGAT-Cre mice in mcherry control and ChR2 groups. The mice were constantly optically stimulated (activation, 473 nm, 20 Hz, and 10 ms duration) every 10 s with 20-s intervals during the whole process. **(D)** The average propofol concentration leads to LORR of mice with optical stimulation of mice in the mcherry control and ChR2 group. **(E)** The RORR times induced by propofol under the optical stimulation of mcherry control and ChR2 group, respectively. **(F)** Representative EEG traces were recorded from mice during propofol-induced anesthesia under the optical stimulation of mcherry control and the ChR2 group. **(G)** The comparison of waveform components in the EEG of mice with an intravenous injection of 20 mg/kg of propofol under ChR2 and mcherry control group. **(H–I)** The average spectral waveform distribution in EEG was recorded from mice under propofol anesthesia under the mcherry control **(H)** and ChR2 group **(I)**. Mean ± SEM. * p < 0.05, ** p < 0.01.

The optogenetic activation of TMN GABAergic neurons affects the conscious state induced by propofol anesthesia was also tested with the AAV-DIO-chR2-mcherry injection in TMN stragety (Figure 4A). Then, the LORR of mice following propofol injection was observed while the anesthesia state of mice was monitored by EEG recording (Figure 4B). Consistent with the chemogenetic results, the propofol dose-response curve in the ChR2 group was also significantly rightward shifted from the curve in the mCherry group (*p* < 0.01) (Figure 4C). Also, there was significant difference in the average propofol concentration, resulting in LORR in half of the mice between the ChR2 and control groups (n = 10, 10.5 ± 0.6 mg/kg vs. 12.3 ± 0.5 mg/kg; *p* = 0.038) (Figure 4D). Meanwhile, compared with the mCherry group, the average RORR time induced by propofol in the ChR2 group decreased by approximately 22 s (n = 9, 283 ± 6.9 s vs. 261 ± 5.8 s; *p* = 0.011) (Figure 4E). Thus, the optical activation of TMN neurons shortens the propofol-induced loss-of-conscious.

The depth of anesthesia was monitored by EEG recording during CSSGA after an intravenous injection of 20 mg/kg propofol. Compared with the mCherry group, the optogenetic activation of the TMN GABAergic neurons resulted in a decrease of δ waves in EEG by approximately 9% (47.7% ± 3% vs. 38.7% ± 3%; *p* = 0.032) and an increase of β waves (14.4% ± 2% vs. 21.8% ± 2.4%; *p* = 0.038) (Figures 4F–I), indicating a reduced anesthesia depth (Figures 4F–I). Thus, the EEG data suggest that the optogenetic activation of TMN GABAergic neurons alters the depth of the anesthesia state during CSSGA induced by propofol. Therefore, enhancing the activity of TMN GABAergic neurons attenuates propofol’s anesthesia effect and shortens the propofol-induced emergence time.

## Discussion

In this study, we revealed that chemogenetic and optogenetic activation of TMN GABAergic neurons attenuated the anesthesia effect of sevoflurane and propofol in mice. The mice with TMN GABAergic activation manifested as rightward-shifted sevoflurane dose-response curves to cause LORR and LOTW, prolonged latency to LORR, and shortened RORR time at a given sevoflurane concentration. In contrast, the chemogenetic inhibition of TMN GABAergic neurons promotes unconscious effects for sevoflurane anesthesia and analgesia, suggesting a crucial role of TMN GABAergic neurons in regulating the arousal state during the sevoflurane-induced anesthesia process.

However, the role of TMN GABAergic neurons in propofol-induced anesthesia differs from the role in sevoflurane-induced anesthesia. The chemogenetic and optogenetic activation of TMN GABAergic neurons attenuated propofol’s loss of consciousness effect, whereas the chemogenetic inhibition of TMN neurons did not alter the RORR time at a given propofol concentration in VGAT-Cre mice. These results suggest the activation of TMN GABAergic neurons also potently promotes arousal in propofol-induced anesthesia, but inhibition of TMN GABAergic neurons did not promote the anesthesia effect of propofol. This phenomenon probably can be explained as the inhibition effect already mimics propofol inhibition’s role. Anyway, these results are consistent with the previous reports that the administration of gabazine (GBZ) in TMN results in attenuated sedation and the activation of histaminergic neurons does not alter the emergence time generated by propofol (Nelson et al., 2002; Zecharia et al., 2012). Thus, our results suggest the vital role of TMN GABAergic neurons in anesthesia.

Previous studies reported the category of histaminergic TMN neurons and projection. Three groups of histamine neurons with different GABA sensitivities have been identified (Sergeeva et al., 2002). Among them, α2-and β3-containing GABA_A_R is most relevant for sleep, whereas the propofol’s action depends on the low expression of the GABA_A_R ε-subunit in the TMN (Rudolph et al., 1999; Sergeeva et al., 2005). The histaminergic neurons in TMN project widely throughout the central nervous system (CNS), including the spinal cord, cerebral cortex, and thalamus (Panula et al., 1989). Notably, the TMN innervated brain regions contain the nuclei involved in the regulation of the sleep-wake cycle, such as the lateral hypothalamus (LH), ventrolateral preoptic nucleus (VLPO), basal forebrain (BF), ventrolateral periaqueductal gray (vlPAG), as well as the monoaminergic nuclei (e.g., VTA, DR, LC) (Yoshikawa et al., 2021). The histaminergic neurons in TMN also receive afferent projections from neurons in the lateral hypothalamus (containing orexin, melanin or neurotensin), the basal forebrain (containing GABA or Ach), dorsal raphe nucleus and locus coeruleus (containing monoaminergic transmitter) (Saper et al., 2005; Yoshikawa et al., 2021). The projection of GABA neurons in VLPO and basal forebrain to TMN probably has been considered the neuronal circuit basis for evaluating TMN as the principal target of propofol anesthesia because propofol exerts its effects through potentiating GABA receptors to modulate hypothalamic sleep pathways (Allada, 2008; Zecharia et al., 2009). But the fact that the removal of synaptic GABA_A_ receptors from histaminergic neurons does not alter the propofol-induced loss of consciousness seems to argue against this idea (Zecharia et al., 2012). In contrast, the antagonized effect on propofol-induced anesthesia by both chemogenetic and optogenetic activation of the TMN GABAergic neurons in our results supports the TMN GABAergic neurons play the promoting arousal role in propofol anesthesia. Therefore, our results provide new insights for understanding the different roles of the activity of the TMN GABAergic neurons in regulating the anesthesia effect of sevoflurane and propofol.

However, the innervation and projection of the GABAergic neurons in TMN were poorly studied. Previous studies rarely use a GABAergic tracer to label the GABAergic projection precisely (Kohler et al., 1985). However, at least we know an atypical GABAergic projection in the cortex is from the tuberomammillary nucleus, which produces atypical tonic inhibition through the extracellular GABAa receptor (Kukko-Lukjanov and Panula, 2003; Melzer and Monyer, 2020). Furthermore, histaminergic cell activation behaviourally promotes wakefulness, whereas GABA release from tuberomammillary projections counteracts this effect (Yu et al., 2015). But this projection is reported from histaminergic neurons in the TMN, which is inconsistent with our results. A recent study stated the GABAergic neurons in TMN may project to the lateral habenula in zebrafish (Ramaswamy et al., 2020). But whether the projection exists in the rat or mice remains unknown. Interestingly, a recent study reported optogenetic activation of LHb glutamatergic neurons produced a hypnosis-promoting effect in isoflurane anesthesia. If these neurons accept the projection from gabaergic neurons from TMN, it probably is a neural circuit that GABAergic activation plays its role in promoting wakefulness (Liu et al., 2021). Thus, the pathway or neural circuits involved in the TMN GABAergic neurons regulating anesthesia effect needs further investigation.

## Data Availability

The original contributions presented in the study are included in the article/supplementary material, further inquiries can be directed to the corresponding authors.
